# Effects of Environmental Endocrine Disruptors on Pubertal Development

**DOI:** 10.4274/jcrpe.v3i1.01

**Published:** 2011-02-23

**Authors:** Samim Özen, Şükran Darcan

**Affiliations:** 1 Pediatric Endocrinology Unit, Mersin Children Hospital, Mersin, Turkey; 2 Department of Pediatric Endocrinology and Metabolism, Ege University School of Medicine, Izmir, Turkey; +90 232 223 07 01+90 324 223 07 22samimozen@gmail.comPediatric Endocrinology and Metabolism Unit, Mersin Children Hospital, 33000, Guneykent, Toroslar, Mersin, Turkey

**Keywords:** puberty, pubertal development, endocrine disruptors

## Abstract

The onset and course of puberty are under the control of the neuroendocrine system. Factors affecting the timing and regulation of the functions of this system may alter the onset and course of puberty. Several environmental endocrine disruptors (EDs) with significant influences on the normal course of puberty have been identified. Numerous animal and human studies concerning EDs have been conducted showing that these substances may extensively affect human health; nevertheless, there are still several issues that remain to be clarified. In this paper, the available evidence from animal and human studies on the effects of environmental EDs with the potential to cause precocious or delayed puberty was reviewed.

**Conflict of interest:**None declared.

## INTRODUCTION

Recent studies have demonstrated a progressive decrease in age of onset of puberty in children around the world. Although the exact reason for this shift is not completely understood, it is generally accepted to be the outcome of a complex interaction between genetic, endocrine and environmental factors. It is also known that owing to the acceleration of industrialization throughout the world, a gradual but significant increase has occurred in the number and amount of environmental pollutants. Some of these environmental pollutants are natural or synthetic chemicals with considerable effects on the endocrine system. The chemicals that have negative effects on the endocrine system are called endocrine disruptors (EDs). EDs exert their effects through different mechanisms: by binding to the relevant hormone receptors; by direct action on cell signaling pathways or on the central nervous system and the neuroendocrine system; by suppression of hormone synthesis; or through their toxic effects on the relevant organs. Several EDs such as phytoestrogens, topical and natural estrogens, pesticides, industrial chemicals and phthalates have been identified as possible agents affecting pubertal development in humans. The potential of EDs to cause precocious puberty has first been noticed in the early 1990s. Afterwards, the effects of EDs on the onset and course of puberty have been demonstrated in numerous animal and human studies and, subsequently, the use  of some of these chemicals has even been prohibited. This paper aims to review the present information regarding environmental EDs that may negatively affect pubertal development. 

**The Effects of Endocrine Disruptors on Puberty**

Recent studies have demonstrated that age of onset of puberty has shifted to younger ages by about 1 or 2 years, while no change has been noted in the age of menarche. The triggering mechanism for the earlier onset of puberty is not clearly understood. It is thought to occur as a result of a complex interaction between genetic, hormonal and environmental factors. Recently, ED chemicals have been intensively accused of being potential hazardous environmental factors (1,2,3,4,5,6). 

EDs are environmental chemicals, which may either be natural or synthetic. Some of the major EDs are presented in Table 1. EDs accumulate in the environment in the long term and are introduced into the human body through water, air, foodstuffs, or through equipments used in the office and home. Additionally, it has been demonstrated that EDs can be transferred from the mother to the fetus via placenta or to the baby via breast milk (1,2,3,4,5).

Due to their hormone-like characteristics, EDs mostly affect the endocrine system in an agonist- or antagonist-specific manner and can be classified according to their mechanisms of action. They influence puberty through their estrogenic, antiestrogenic, androgenic, antiandrogenic effects or through their direct effects on the gonadotropin-releasing hormone (GnRH). These chemicals may exert their estrogenic effects either directly by binding to estrogen receptors, increasing aromatase activity and increasing estrogen sensitivity or indirectly by their effect on GnRH, leading to an increase in endogenous estrogen production.  All of these effects may result in precocious puberty. EDs produce antiestrogenic and androgenic effects through inhibition of aromatase enzyme activity and steroidogenic enzyme production. They display antiandrogenic effects via suppression of testicular steroidogenesis and androgen-receptor blockade. Thus, depending on their mechanism of action, EDs may lead to precocious puberty, to delayed puberty, or to sexual differentiation disorders (1,4,5,6,7,8,9,10,11). Classification of some of the EDs according to their mechanism of action is presented in Table 2.

**Natural Endocrine Disruptors**

The best known chemicals in this group are phytoestrogens, which are relatively weak compared to endogenous estrogen. They are found in several nutrients that are  frequently consumed in daily life (i.e. carrots, garlic, apple, coffee, cherry, parsley, legumes). Phytoestrogens have estrogenic effects when consumed in huge amounts and antiestrogenic effects at low concentrations (4,7,8). 

**Synthetic Endocrine Disruptors**

Diethylstilbestrol (DES) is the best known ED with strong estrogenic activity. It was first synthesized in 1938 and has since been widely used worldwide for medical zindications including pregnancy toxemia and preterm labor. However, a twofold increase in breast cancer incidence was observed in mothers exposed to DES. It was also shown that the incidence of cervical cancer, ovarian germ cell cancer, cervical or vaginal dysplasia, and vaginal clear-cell adenocarcinoma was increased in female infants born to mothers exposed to DES (12). The production or marketing of this chemical is prohibited since 1997. 

Many chemicals including pesticides, fungicides, herbicides h_2_o used in agriculture, cleaning substances used in daily life, contents of cosmetic products, dyes, plastic substances and solvents are likely to be EDs. As neutralization or inactivation is difficult and most of these substances often accumulate in fat tissue, they may persist in the body for long periods of time and cause harmful effects (4,5,6,7,8,9,10,11). Time of exposure to EDs is important in terms of their detrimental effects. Male rodents exposed to 2,3,7,8-tetrachlorodibenzo-p-dioxin (TCDD) in intrauterine life were found to experience problems in masculinization of internal and external genitalia, descent of testicles, androgen production and in spermatogenesis. On the other hand, it was observed that postnatal exposure to TCDD was associated only with impairment of spermatogenesis and somatic and genital growth. The dose and duration  of exposure to EDs is also important in terms of potential negative consequences. The negative effects may become more serious as the duration of exposure and dose increase (1,11,13). 

EDs do not necessarily lead to similar effects in all situations. For example, phytoestrogens have estrogenic effects in high doses, while they exert antiestrogenic effects in low doses (1,7). 

**Studies on Role of EDs in Precocious Puberty **

Several animal and human studies have been conducted investigating the role of EDs in precocious puberty. EDs may cause early puberty via their estrogenic or antiandrogenic effects and also by increasing GnRH production (1,4,5,6,7,8,9,10,11). 

**Animal Studies**

EDs have first been noted in experimental studies and gained significance through evidence from animal experience. Following the accidental spill of high amounts of dichlorodiphenyltrichloroethane (DDT) and dicofol from a chemical company into Lake Apopka in Florida in 1980s, it has been noted that male alligators living in the lake had smaller phalluses, reduced serum testosterone levels and abnormal gonadal structures concurrently with high serum DDT levels (14). It was observed that administration of methoxychlor, a pesticide with potential estrogenic effects, in female rats during pregnancy and lactation periods, led to vaginal opening and appearance of puberty at a very early age in their female offspring (15). Similarly, accelerated and early vaginal opening was noted in female rats following subcutaneous injections of bisphenol A (BPA), genistein, resvetarol, zearalenone or DES in the prenatal period, findings confirming the estrogenic effects of these chemicals (16,17). Administration of methoxychlor or 17 beta-estradiol after the cessation of breastfeeding also resulted in early vaginal opening in female rats (15,17). In addition, Markey et al (18) showed that prenatal exposure to BPA caused early growth and proliferation of breast tissue as well as increased susceptibility to endogenous estrogen in female rats. Methoxychlor, an antiandrogenic and estrogenic agent, and also vinclozolin, an antiandrogenic agent, were shown to lead to delayed puberty in male rats and precocious puberty in female rats (19).

Dioxins are environmentally toxic chemicals. TCDD is a type of dioxin, which exerts its estrogenic or antiandrogenic effects by causing alterations in gene expression via binding to aryl hydrocarbon receptors. In utero, exposure of rodents to even low doses of this substance was found to result in reproductive system abnormalities and precocious puberty findings in the females and delayed puberty in the males (7,8,9,10,11,20,21,22). Other chemicals that can produce the same effects by binding to aryl hydrocarbon receptors include industrial products such as polychlorinated biphenyls (PCBs), polybrominated diphenyl ether and the pesticide methoprene (1,7,8,9,10,11). 

Some of the environmental chemicals may impair neuroendocrine functions through their effect on the central nervous system and the hypothalamic-hypophyseal-gonadal (HHG) axis. These include pesticides such as thiram, molinate, metam sodium, chlordimeform, amitraz, triazoles, dichloroacetic acid, atrazine, propazine, simazine, methanol and linuron (1). It has been shown in a study on rats that atrazine causes delayed puberty by suppressing luteinizing hormone (LH) and prolactin levels (23,24,25). In rats prenatally exposed to BPA, an increase in estrogen feedback as well as development of precocious puberty via inhibition of tyrosine hydroxylase activity in rostral preoptic periventricular neurons have been observed (26). Howdeshell et al (27) also noted precocious puberty in rats prenatally exposed to BPA. 

Some of the EDs affect puberty by inhibiting the synthesis of endogenous hormones such as testosterone, 17 beta-estradiol and adrenal steroids via competitive inhibition of P450 steroidogenic enzymes (C17,20-lyase, aromatase) (1). Delayed puberty has been reported by exposure to imidazole group fungicides, ketoconazole and fadrozole in the peripubertal period (28). Another pesticide, prochloraz, suppresses estrogen and androgen synthesis via inhibition of aromatase and 17,20-lyase (29,30).

**Studies in Humans**

Studies have shown that several environmental chemical pollutants including DDT/ dichlorodiphenyldichloroethylene (DDE), PCBs, polybrominated biphenyls (PBB), 

hexachlorobenzene, endosulfan, dioxins, heavy metals and phthalates affect puberty in humans (1,2,3,4,5,6,7,8,9,10,11).

Following the pollution of the environment by PBB as a result of an industrial accident in the state of Michigan in 2000, Blanck et al (31,32) investigated the serum PBB levels in pregnant women and compared ages of puberty onset and menarche in their daughters who were or were not breast-fed. When the girls with intrauterine exposure to high PBB concentrations (>7 ppm) were compared with the girls who had no exposure (<1 ppm), menarche was observed to occur one year earlier in those who were exposed to high concentrations. Among those exposed to high concentrations, breast-fed girls were observed to have pubarche one year earlier than the girls who were not breast-fed. No significant difference in terms of breast development was reported in this study. Gladen et al (33) found a relationship between intrauterine exposure to high doses of these pesticides and early thelarche and early pubarche in girls.

Effects of DDT, with proven estrogenic effect, and its metabolite DDE on pubertal development have been investigated in several studies. After their detrimental effects had been explicated, DDT and its metabolites, formerly used widely as pesticides in agriculture, were banned in many countries. Its use has also been forbidden in Turkey since 1985. Vasiliu et al (34) reported that menarche occurred 1 year earlier in girls exposed to high amounts of DDT/DDE in the intrauterine period. Krstevska-Konstantinova et al (35) found that precocious puberty was 80 times more frequent and serum DDE levels significantly higher in daughters of immigrants compared to native Belgians. An association between serum DDT/DDE concentrations and early menarche was reported in a study conducted in Chinese textile workers (36).

Methoxychlor is an organochlorine widely used as a pesticide in agriculture. It was shown to impair reproductive behavior and functions in male rats due to its estrogenic effect (37). There are no human studies on the effects of this pesticide. Methoxychlor, which is a chlorinated hydrocarbon pesticide and has been introduced for use instead of DDT, accumulates in very little quantities in fat tissue. Although it has been reported to produce estrogenic effects similar to DDT and its metabolites in animal studies, there are no studies on the effects of this pesticide on precocious puberty in humans (15,38,39).

Endosulfan and its derivatives are pesticides widely used in agriculture and are thought to have antiandrogenic and estrogenic effects (1,2,3,4,5,6,7,8,9,10,11). In animal studies, endosulfan was reported to have an estrogenic effect and to cause inhibition of follicle-stimulating hormone (FSH), LH and testosterone production  (40,41). In an Indian study, LH was found to be increased and testosterone levels decreased in men exposed to endosulfan (41).

In a recent Danish study, as compared to the normal population, significantly earlier thelarche in girls and more frequent genital abnormalities such as hypospadias, micropenis in boys were noted in children of greenhouse owners, and it was suggested that these findings were due to pesticides. However, no pesticide analysis was conducted in this study (42). Antiandrogenic effect of vinclozolin has been shown in male animals (43). Although the estrogenic effect of this chemical used widely in agriculture is not known for sure, it has been reported that it might have an antiandrogenic effect and produce an estrogen-like effect through stimulation of estrogen receptor a (44).

The effects of PCB exposure on puberty have also been investigated by several authors. There are several sub-types of this group of substances that are thought to have estrogenic effects (1,2,3,4,5,6,7,8,9). While no relationship was detected between intrauterine and/or postnatal exposure to PCB and age of puberty or menarche in some studies, it was reported in others that menarche occurred significantly earlier in girls who were exposed to PCB 52, 70, 101, +90 and 187 sub-groups (45,46).

Chlorine is used as a whitener in many solutions used in daily life. Dioxins are formed as by-products of incomplete combustion of chlorinated waste products. Contact of plastic products with hot surfaces (serving hot liquids or food in plastic glasses or plates, microwave use) may also lead to production of dioxins. Exposure of humans to huge amounts of dioxin is only possible in industrial accidents. However, exposure to very small amounts may occur due to the contact of several daily used substances such as plastic glasses, plates, toys, cleaning substances or paper whitened by chlorine with hot surfaces. Dioxins can be taken by animals through ingestion of contaminated soil, vegetables and water, and due to long-term adipose tissue accumulation, they may be transferred to humans from animal meat or milk (1,2,3,4,5,6,7,8,9,10,11). There are two human studies investigating this toxic substance, which has been found to be associated with estrogenic effect and precocious puberty in animal studies. In a Belgian study, Den Hond et al (47) observed a reduction in testicular volume in boys exposed to a substance with dioxin-like activity, but failed to note a change in pubertal development. In this same study, the authors reported a delay in the breast development in girls, but no change in age of menarche or pubarche. In a study on Italian girls, Warner et al (48) found no change in age of menarche in girls exposed to TCDD postnatally before the age of 5 years. Mothers of these children were not exposed to this substance during pregnancy or before. 

Colon et al (49) observed significantly higher levels of phthalate esters in 31 Puerto Rican girls with early thelarche compared to controls. Phthalates are used as plastic softeners and as preservatives in some cosmetic products and they may be found in plastic toys, hair sprays, deodorants, shampoo, nail polish and perfumes, and may easily enter the human body (1,2,3,4,5,6,7,8,9,10,11). The use of these substances, which possess estrogenic and antiandrogenic properties, has been prohibited in many European countries.

Lead, a heavy metal and one of the major environmental pollutants, has also been found to affect puberty. It was observed that menarche and pubarche were delayed in girls with high serum lead levels (50,51). 

In addition to its teratogenic and cancerogenic features, BPA, which is found in huge amounts in polycarbonate plastics (i.e. baby feeding bottles), has also been suggested to have estrogenic effects causing precocious puberty. Although BPA has been investigated thoroughly in many animal studies, human studies are lacking. It has been suggested in some studies that exposure to BPA at early ages is associated with an increased incidence of breast cancer (52).

**Table 1:**
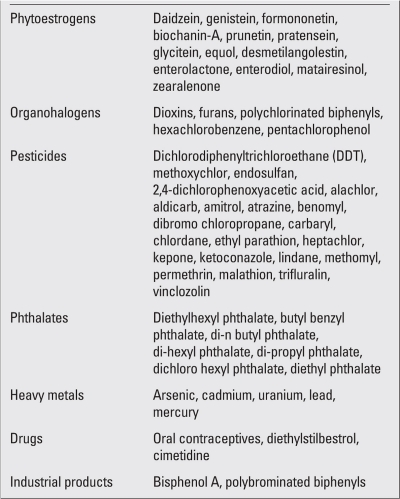
Major endocrine disruptors

**Table 2:**
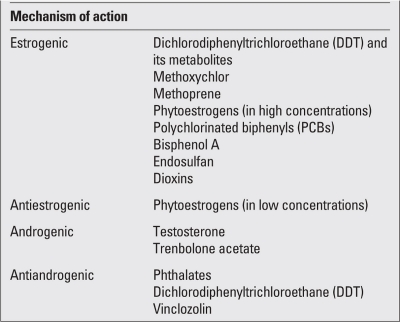
Classification of some of endocrine disruptors according to their mechanism of action

## CONCLUSION

In conclusion, it is worth noting that for EDs, to cause impairment of endocrine functions, time of exposure is as important as dose, duration and age at exposure. Moreover, a single chemical substance may result in multiple endocrine system dysfunctions via several mechanisms. To date, various EDs have been shown to exert unwanted effects on pubertal development. It is possible that there are numerous other chemicals with potential harmful effects on pubertal development. Future population-based studies are warranted for further investigation.
